# Diagnosis of Sporadic Creutzfeldt-Jakob Disease in Both Kidney Recipients From the Same Donor: Was It Graft Transmission?

**DOI:** 10.7759/cureus.105229

**Published:** 2026-03-14

**Authors:** Maciej M Ponczek, Zuzanna Jakubowska, Ewa Wojtaszek, Jolanta Malyszko

**Affiliations:** 1 Department of Nephrology, Dialysis and Internal Medicine, Medical University of Warsaw, Warsaw, POL

**Keywords:** creutzfeldt-jakob disease, donor transmission, kidney transplantation, prion disease, prion transmission

## Abstract

Recognizing rare causes of neuropsychiatric symptoms in transplant recipients can be challenging, as neurological complications occur in many patients and are usually due to infections, comorbidities, or drug toxicity. Once these factors have been ruled out, prion diseases such as Creutzfeldt-Jakob disease (CJD) should be considered. CJD can spread through medical interventions. Current diagnostic criteria for iatrogenic CJD take into account the possibility of transmission through corneal or dural transplants, contaminated neurosurgical tools, as well as administration of cadaveric pituitary hormones. They do not recognize solid organ transplantation as a viable transmission pathway. We present a unique case of concurrent rapidly progressive dementia in both kidney recipients from the same donor, in one of whom the diagnosis of CJD was confirmed. The clinical course, CSF findings, and neuropathology suggested possible prion transmission from their common donor. This case raises awareness of the possibility of transplant-derived prion infections and illustrates the complexity of the diagnostic workup. We advocate for wider adoption of appropriate diagnostic tools.

## Introduction

Diagnosing rare causes of neuropsychiatric symptoms in transplant recipients is challenging, as approximately a third of this population develops neurologic alterations [1]. These are often related to comorbidities, drug toxicity, or opportunistic infections. Therefore, it is vital to exclude these before considering less common etiologies such as prion diseases.

Creutzfeldt-Jakob disease (CJD) is a rare, progressive, neurodegenerative prion disease affecting approximately one to two individuals per million [2]. It is caused by a pathogenic, misfolded isoform of cellular prion protein (PrP^C^), acting as a template that promotes further conversion of normal PrP^C^. A total of 85-95% are cases of sporadic Creutzfeldt-Jakob disease (sCJD), 5-15% are genetic, and less than 1% are acquired cases, classified as iatrogenic Creutzfeldt-Jakob disease (iCJD) or variant Creutzfeldt-Jakob disease (vCJD). CJD is clinically heterogeneous. Rapid neuropsychiatric decline with profound dementia, accompanied by neurological symptoms, such as nystagmus, ataxia, spasticity, hypokinesia, and dystonia, is frequently observed. The disease leads to akinetic mutism and death usually within one year of symptom onset [3]. A definite diagnosis requires neuropathological, immunocytochemical, or biochemical confirmation. Most cases are designated as probable CJD based on clinical features, magnetic resonance imaging (MRI), typical electroencephalography (EEG) patterns, cerebrospinal fluid (CSF) biomarkers, and real-time quaking-induced conversion (RT-QuIC) [4].

iCJD was historically caused by corneal or dural transplants [5,6], contaminated neurosurgical tools [7], as well as administration of cadaveric pituitary hormones [8]. Reported incubation periods range from one to over 40 years [9]. Thanks to current practices, iCJD is believed to have been largely eradicated [10]. In the case of vCJD, transmission through infected blood products is possible [11]. The few described cases of CJD following an organ transplant are thought to be sCJD, developed incidentally after the procedure. Here, we present a case of concurrent rapidly progressive dementia in both kidney recipients from a common donor, one of whom was diagnosed with definite sCJD. Our aim is to showcase the difficult diagnostic workup for prion infection and emphasize the findings that should prompt clinicians to consider CJD.

## Case presentation

Case 1

A 51-year-old male patient with a history of kidney transplantation four months prior presented with a fever of unknown origin, dry cough, headache, and general weakness that had persisted for a week. Additionally, the patient was experiencing dizziness and hand tremors, believed to be secondary to the immunosuppressive treatment. His vital signs were normal with insufficiently controlled hypertension. He was fully oriented. On initial neurological examination, he was slow to respond and exhibited bilateral asymmetric ptosis. Pathological reflexes were absent, but the finger-nose test was abnormal on both sides. The patient was admitted to the department of nephrology for testing.

His past medical history involved renal cell carcinoma in his right kidney treated with a nephrectomy, and subsequent unspecified glomerulonephritis in the remaining kidney, leading to end-stage kidney disease. The patient had been on dialysis for over three years before the kidney transplant. The donor was a 48-year-old male with a history of alcohol and methamphetamine abuse and intracranial bleeding. He was cytomegalovirus (CMV) IgG-positive. The donor had HLA-A*1,3-B*7,8-C*7-DRB1*17,15, whereas the recipient had HLA-A*3,11-B*7,51-C*7,15-DRB1*4,15, with three HLA mismatches. The post-transplant period was complicated by delayed graft function and an incisional hernia. The recipient was then diagnosed with a CMV infection, which was successfully treated with ganciclovir and the discontinuation of mycophenolate mofetil. Since then, the patient has stayed on prednisone and tacrolimus immunosuppression. Graft function had been satisfactory.

Following the patient's admission, suspicions of neuroinfection or drug neurotoxicity were raised. Tacrolimus and prednisone were changed to methylprednisolone as the sole immunosuppressant, and ganciclovir was administered. No improvement in the patient’s neurological symptoms was observed in the following days. His C-reactive protein, procalcitonin, and blood count remained normal. Blood and urine cultures were taken. Serologic tests for *Toxoplasma gondii* IgM and IgG and *Borrelia burgdorferi* were performed, and all were negative. Cerebrospinal fluid was collected, which revealed an elevated total protein of 84.7 mg/dL (normal range: 15-45 mg/dL) and a nucleated cell count of 30/µL (normal range: ≤5/µL). A quantitative polymerase chain reaction neurological panel indicated CMV in the CSF, prompting an increase in ganciclovir doses. Infections with enterovirus, herpes simplex virus type 1 (HSV-1), herpes simplex virus type 2 (HSV-2), human herpesvirus 6 (HHV-6), varicella-zoster virus (VZV), adenovirus, *Cryptococcus neoformans*, hepatitis B virus (HBV), hepatitis C virus (HCV), and syphilis were ruled out.

A week after the patient’s admission, a marked cognitive decline was noticeable. Gait instability and weakened muscle strength were observed. He started exhibiting a positive right-sided Babinski sign and developed dysphagia, requiring a pureed diet.

The following week, his speech deteriorated, and he responded with singular words and subsequently only monosyllables. The patient became incontinent and unable to stand or walk. He retained the ability to recognize personnel and his family and to localize the source of pain. A few days later, he stopped opening his eyes in response to a voice. His pupils remained reactive, and his breathing was normal. Further diagnostics revealed no tangible cause of symptom exacerbation, and the patient was transferred to the intensive care unit (ICU) with a Glasgow Coma Scale (GCS) score of 9/15 (E2-V2-M5). In the ICU, increased muscle rigidity was observed despite regular rehabilitation.

In the next few days, the patient improved to a GCS of 12/15 (E4-V2-M6), although his mental state fluctuated. The decision was made to transfer him back to the department of nephrology, where, in three days, the patient’s GCS decreased to 8/15 (E1-V2-M5). On renewed neurological examination, the patient established no verbal communication but followed simple commands, such as to show his tongue. Persistent upper extremity flexion and spasticity were notable.

Given the patient’s immunosuppressed state, progressive multifocal leukoencephalopathy (PML) was considered. Further virological testing was repeated, which ruled out the John Cunningham (JC) virus, tick-borne encephalitis, and rabies.

**Figure 1:**
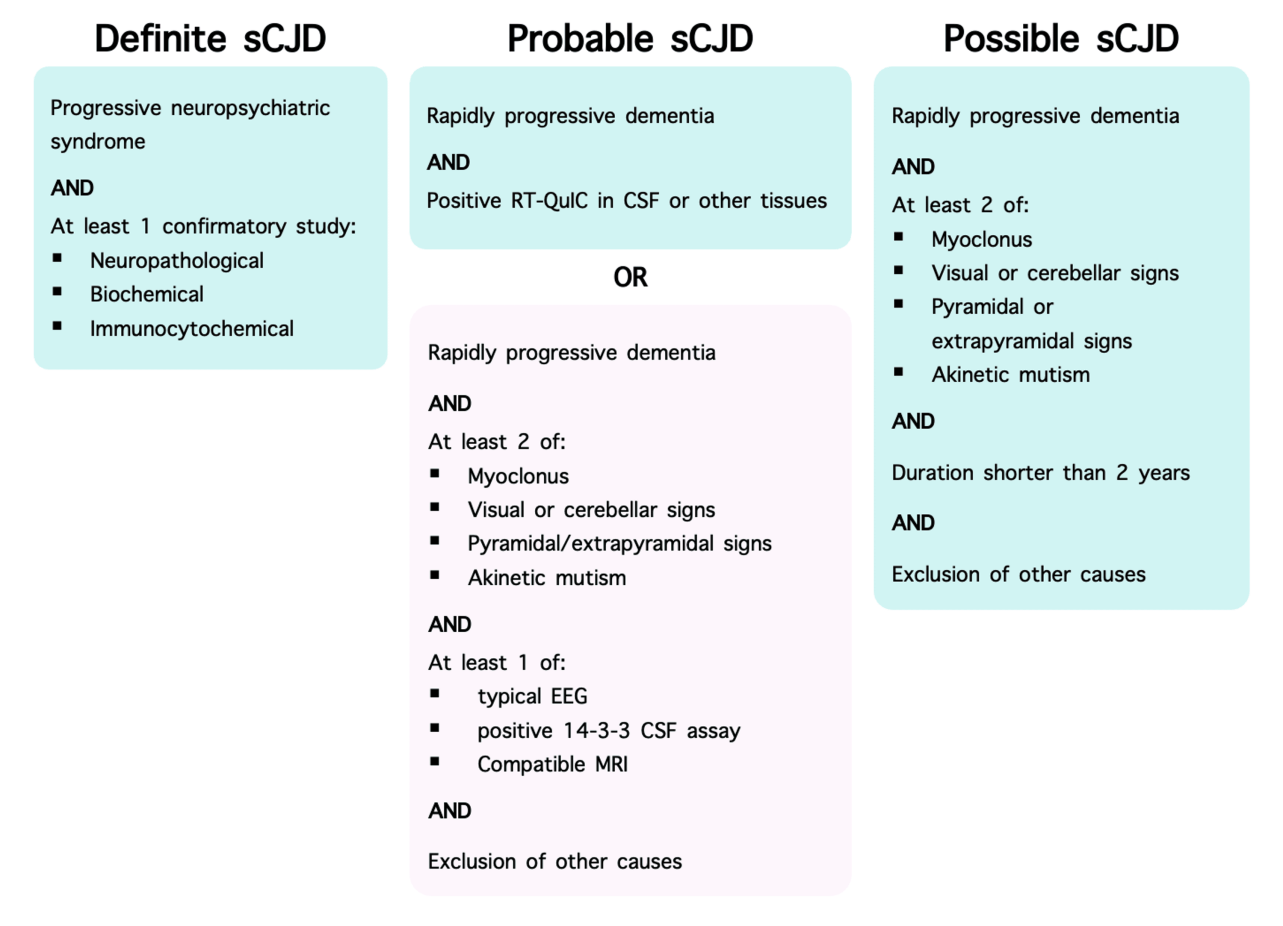
Diagnostic criteria for sporadic Creutzfeldt-Jakob disease. The set of criteria used to diagnose the male patient with probable CJD is marked in purple. Adapted from the Centers for Disease Control and Prevention [12]. CJD: Creutzfeldt-Jakob disease; sCJD: sporadic Creutzfeldt-Jakob disease; RT-QuIC: real-time quaking-induced conversion; CSF: cerebrospinal fluid; EEG: electroencephalography; MRI: magnetic resonance Imaging.

A suspicion of a prion disease was raised; thus, CSF 14-3-3 protein assay was ordered, which was positive. Due to the exclusion of other potential etiologies, a diagnosis of probable sCJD was initially made. The patient’s rapidly progressive dementia, positive 14-3-3 assay, together with his persistent ataxia (abnormal finger-nose test) and pyramidal signs (spasticity), have met probable sCJD criteria (Figure 1). The highly specific RT-QuIC assay would have allowed for an additional confirmation or exclusion of sCJD in the male patient. Unfortunately, it could not be performed due to its limited availability in Poland, where the patient was treated.

**Figure 2:**
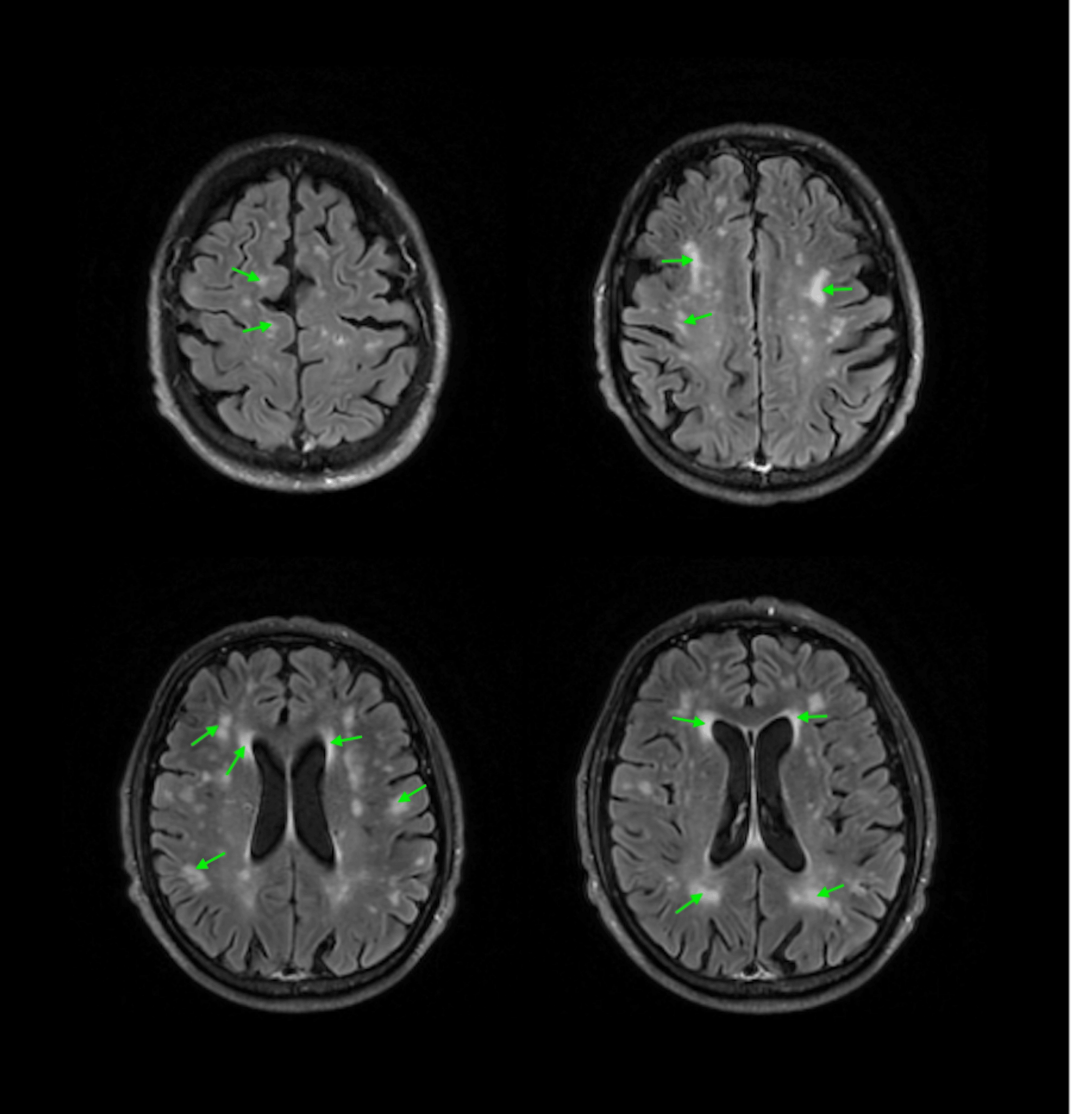
Fluid-attenuated inversion recovery (FLAIR) MRI showed multiple, partially confluent foci and hyperintense areas (marked with arrows) of various sizes located in the white matter of both cerebral hemispheres. Bilateral widening of the Virchow-Robin perivascular space was present. Notably, T1-weighted images showed normal signal intensity without focal lesions; diffusion-weighted imaging (DWI) sequence and corresponding apparent diffusion coefficient (ADC) maps revealed no evidence of restricted diffusion. The patient’s MRI findings were not consistent with the diagnosis of CJD. Typical MRI diagnostic features of this disease involve high intensity of the caudate nucleus, putamen, or at least two cortical regions (temporal, parietal, occipital), either on DWI or FLAIR sequences [13].

The patient’s MRI (Figure 2), while not pointing to a specific diagnosis, did not support this designation, as white matter hyperintensities are not a hallmark of CJD, apart from a fringe panencephalopathic disease phenotype. However, specific imaging was not required to meet the criteria for probable sCJD. Nevertheless, there remained uncertainty regarding the validity of the diagnosis.

Weeks after the initial diagnosis, the progression of the patient’s symptoms ceased, which contributed to the diagnostic uncertainty. Following extensive neurological and radiological consultations, the decision was made to discontinue diagnostic escalation, as available options had been exhausted. Since then, the patient has been treated conservatively.

The disease has led to an irreversible cognitive and functional impairment in the patient, who remains under the care of his family, due to the unavailability of dedicated care centers. He has been able to follow simple commands and perform basic daily activities. Speech difficulties have persisted. He has been receiving regular outpatient care from the department of nephrology for 14 months since hospital discharge, which is compatible with the diagnosis of sCJD. Despite continued immunosuppressive monotherapy with corticosteroids, the patient’s graft function remains satisfactory and stable, and he has been dialysis-free.

Case 2

Concurrently, the other recipient of a kidney from the common donor was hospitalized at the department of nephrology. The 52-year-old female presented with a seven-day history of tinnitus and subsequent left-sided deafness. No etiology could be established as she developed profound dementia, near-total deafness, diplopia, horizontal nystagmus, difficulties speaking, and a bilateral Babinski sign in a matter of eight days. On day 15 post-admission, she required mechanical ventilation. She was treated with intravenous polyclonal antibodies for suspected autoimmune encephalitis, which proved unsuccessful. Diagnostic and treatment attempts continued as she became entirely immobile and developed diabetes insipidus. She died on day 34 after admission to a neurological ICU.

The decision was made to determine her definitive diagnosis with neuropathology. The histopathological report described specific brain lesions, and a definitive diagnosis of CJD was established.

## Discussion

A diagnosis of iCJD can be made in sCJD patients in the presence of a recognized risk of exposure, that is, neurosurgical procedures utilizing dural or corneal grafts, as well as performed with contaminated instruments, and the use of cadaveric human pituitary hormones. Evidence of a possible link between CJD and other surgical procedures, such as kidney transplantation, is conflicting and based on limited, low-quality studies [14-17]. Only three cases of CJD following a solid organ transplant were previously reported (Table 1) [18-20]. They lacked evidence suggesting the transmission of prions with the transplant and were presumed to have been de novo sCJD cases.

**Table 1:** A summary of previously reported cases of CJD developed following a solid organ transplantation. CJD: Creutzfeldt-Jakob disease.

Year of transplantation	Transplanted organ	Patient's age at diagnosis	Patient's sex	Time between transplantation and diagnosis of CJD	Study type
2018	Kidney	71	Female	2 years	Case report
Not specified (1996-2014)	Liver	57	Female	5 years	Retrospective epidemiological study
1991	Liver	57	Female	2 years	Case report

Given the infectious character of the disease, the possibility of iCJD through an unusual graft transmission of prions from the common donor was initially considered for the two patients described in this report. Further inquiry, however, proved this unlikely. Even assuming the accuracy of the male patient’s CJD diagnosis and considering that both patients developed this disease within a remarkably similar timeframe, several factors suggest a low probability of prion transmission with the kidney grafts. Firstly, the incubation period of only four months is incompatible with the incubation times reported in cases of iCJD, which range from one to over 40 years. Secondarily, the diagnosis could not be established for the donor, as no routine screening for prions is done, and no specimens remained to be assessed. Furthermore, kidney concentrations of prions are extremely low when compared to the brain, which has been the usual source of contamination in iCJD, making the probability of kidney graft transmission even less likely. All these reasons support the designation of the female patient’s histopathologically confirmed CJD as sporadic.

For the male patient, great diagnostic efforts did not make it possible to establish a definite cause. The complex differential diagnosis of his rapidly progressive neuropsychiatric symptoms involved etiologies typical for the general population (autoimmune encephalitis, neurodegenerative diseases, and viral, bacterial, and fungal infections), as well as those specifically reported in transplant recipients (neurotoxicity caused by calcineurin inhibitors, PML, toxoplasmosis, *Cryptococcus neoformans*, CMV, primary central nervous system lymphoma). All of these have been ruled out, and probable sCJD was determined as the most plausible diagnosis, in accordance with the diagnostic criteria. Nevertheless, a reasonable uncertainty about the diagnosis persists, as the patient’s imaging studies were not characteristic of CJD. His symptoms and their progression, broadly consistent with rapidly progressive dementia in CJD, were not entirely specific for this disease.

**Figure 3:**
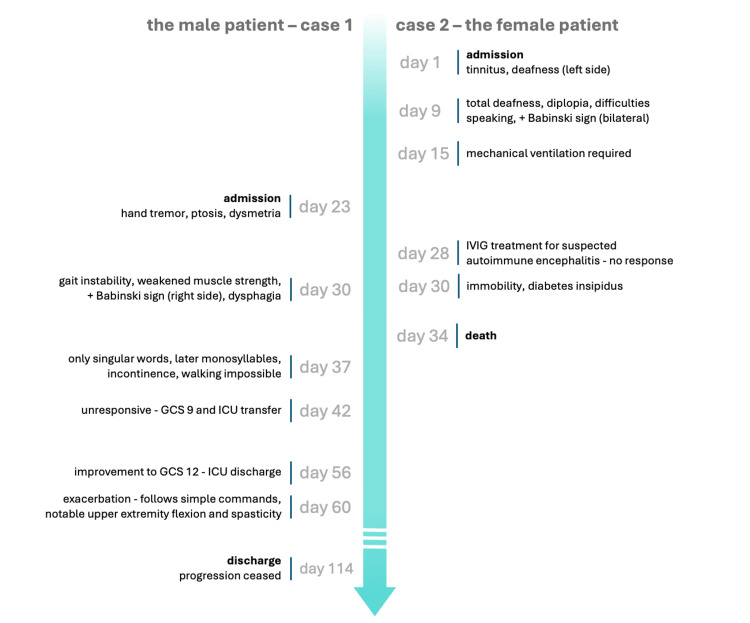
Timeline (top to bottom) of the most important neurological alterations in the course of both patients’ hospitalizations. The male patient’s timeline is on the left of the axis, and the female patient’s is on the right. The days are numbered starting from the female patient’s hospital admission (day 1). This timeline is meant to showcase the rapid progression of both patients as well as the parallel symptom onset four months after the two patients' kidney transplantation. GCS: Glasgow Coma Scale; ICU: intensive care unit; IVIG: intravenous immunoglobulin.

In contrast, the female patient’s presentation was rather characteristic of sCJD. Most of her symptoms are often observed in sCJD patients regardless of the exact clinical subtype of the disease. Their presence, alongside rapid neuropsychiatric decline spanning at most two years, should prompt a clinician to consider sCJD diagnosis. Symptoms depend on the predominant involvement of individual brain regions and typically involve cortical dysfunction (memory impairment, mood changes, visual hallucinations, apraxia, aphasia), myoclonus, cerebral symptoms (ataxia, nystagmus), pyramidal (Babinski sign, spasticity), and extrapyramidal signs (rigidity, dystonia). The female patient presented most of these at various points of her short disease course (Figure 3). However, one of her prominent symptoms, hearing loss, is not a typical sCJD finding. Sensory deficits are more frequently associated with vCJD [21].

The female patient’s later course was marked by a short period of immobility, which ensued just four days before her death. The rapid progression made it impossible to determine whether that state could be ascribed to akinetic mutism, although it is strongly implied by the patient’s subsequent histopathological diagnosis of CJD. Akinetic mutism is a state of preserved alertness with the inability to initiate voluntary movement or speech, typical for end-stage sCJD. Differential diagnosis must primarily include catatonia, locked-in syndrome, non-convulsive status epilepticus, hypoactive delirium, stroke, abulia, and depression.

Another patient in our center had received a liver transplant from that same donor. He undergoes regular checkups and, as of writing this report, presents no signs of CJD.

## Conclusions

Our case report documents one of the very rare instances of patients developing CJD following a transplantation of an organ not typically recognized as a source of prion contamination. Detailed analysis of this occurrence did not provide strong evidence of transmission with the kidney graft, despite the unusual temporal association in both patients. Nevertheless, potential instances of transplant-associated infections warrant thorough reporting, as seeking possible transmission pathways of uncertain significance is vital for the implementation of new preventative public health measures.

Detection of key clinical signs of neurocognitive decline and early diagnosis are vital for immunocompromised patients, such as transplant recipients. Optimal diagnostic tools, allowing for early diagnosis and reliable differentials, should be easily accessible. We advocate for wider adoption of advanced diagnostic tools like RT-QuIC, which should be incorporated in suspected prion disease cases.

## References

[1] Senzolo M, Ferronato C, Burra P (2009). Neurologic complications after solid organ transplantation. Transpl Int.

[2] Klug GM, Wand H, Simpson M (2013). Intensity of human prion disease surveillance predicts observed disease incidence. J Neurol Neurosurg Psychiatry.

[3] Haywood AM (1997). Transmissible spongiform encephalopathies. N Engl J Med.

[4] Watson N, Hermann P, Ladogana A (2022). Validation of revised International Creutzfeldt-Jakob disease surveillance network diagnostic criteria for sporadic Creutzfeldt-Jakob disease. JAMA Netw Open.

[5] CDC CDC (1997). Creutzfeldt-Jakob disease associated with cadaveric dura mater grafts -- Japan, January 1979-May 1996. MMWR Morb Mortal Wkly Rep.

[6] Heckmann JG, Lang CJ, Petruch F, Druschky A, Erb C, Brown P, Neundörfer B (1997). Transmission of Creutzfeldt-Jakob disease via a corneal transplant. J Neurol Neurosurg Psychiatry.

[7] Johnson RT, Gibbs CJ Jr (1998). Creutzfeldt-Jakob disease and related transmissible spongiform encephalopathies. N Engl J Med.

[8] Brown P, Preece MA, Will RG (1992). "Friendly fire" in medicine: hormones, homografts, and Creutzfeldt-Jakob disease. Lancet.

[9] Knight R (2017). Infectious and sporadic prion diseases. Prog Mol Biol Transl Sci.

[10] Brown P, Brandel JP, Sato T (2012). Iatrogenic Creutzfeldt-Jakob disease, final assessment. Emerg Infect Dis.

[11] Llewelyn CA, Hewitt PE, Knight RS, Amar K, Cousens S, Mackenzie J, Will RG (2004). Possible transmission of variant Creutzfeldt-Jakob disease by blood transfusion. Lancet.

[12] (2026). CDC. Clinical overview of Creutzfeldt-Jakob disease (CJD). https://www.cdc.gov/creutzfeldt-jakob/hcp/clinical-overview/index.html.

[13] Collie DA, Sellar RJ, Zeidler M, Colchester AC, Knight R, Will RG (2001). MRI of Creutzfeldt-Jakob disease: imaging features and recommended MRI protocol. Clin Radiol.

[14] Collins S, Law MG, Fletcher A, Boyd A, Kaldor J, Masters CL (1999). Surgical treatment and risk of sporadic Creutzfeldt-Jakob disease: a case-control study. Lancet.

[15] de Pedro-Cuesta J, Mahillo-Fernández I, Rábano A (2011). Nosocomial transmission of sporadic Creutzfeldt-Jakob disease: results from a risk-based assessment of surgical interventions. J Neurol Neurosurg Psychiatry.

[16] Ward HJ, Everington D, Cousens SN (2008). Risk factors for sporadic Creutzfeldt-Jakob disease. Ann Neurol.

[17] Ward HJ, Everington D, Croes EA (2002). Sporadic Creutzfeldt-Jakob disease and surgery: a case-control study using community controls. Neurology.

[18] Créange A, Gray F, Cesaro P (1995). Creutzfeldt-Jakob disease after liver transplantation. Ann Neurol.

[19] Molesworth A, Yates P, Hewitt PE, Mackenzie J, Ironside JW, Galea G, Ward HJ (2014). Investigation of variant Creutzfeldt-Jakob disease implicated organ or tissue transplantation in the United Kingdom. Transplantation.

[20] Sharma N, Wang L, Namboodiri H (2022). Creutzfeldt-Jakob disease following kidney transplantation. Cureus.

[21] Heath CA, Cooper SA, Murray K (2011). Diagnosing variant Creutzfeldt-Jakob disease: a retrospective analysis of the first 150 cases in the UK. J Neurol Neurosurg Psychiatry.

